# New Therapies for the Management of Chronic Kidney Disease

**DOI:** 10.7759/cureus.81824

**Published:** 2025-04-07

**Authors:** Andrew M Treihaft, Manish A Parikh, Kaedrea A Jackson, William H Frishman, Stephen J Peterson

**Affiliations:** 1 Department of Medicine, New York-Presbyterian Brooklyn Methodist Hospital, Brooklyn, USA; 2 Department of Medicine, Weill Cornell Medicine, New York, USA; 3 Department of Emergency Medicine, New York-Presbyterian Brooklyn Methodist Hospital, Brooklyn, USA; 4 Department of Emergency Medicine, Weill Cornell Medicine, New York, USA; 5 Department of Medicine, New York Medical College, Valhalla, USA

**Keywords:** ace inhibitors, aldosterone, chronic kidney disease, mineralocorticoid receptor antagonists, sglt2 inhibitors

## Abstract

A major public health concern gripping the nation is chronic kidney disease (CKD), and for individuals concomitantly diagnosed with type 2 diabetes mellitus (T2DM), the coexistence significantly increases the cardiovascular morbidity and mortality by two to three times higher than patients diagnosed without CKD. CKD management encompasses both non-pharmacological approaches, such as dietary sodium restriction and lifestyle modification for blood pressure control, and pharmacological approaches. Current pharmacological management focuses on four key pillars: renin-angiotensin system inhibitors (RASi), sodium-glucose cotransporter-2 inhibitors (SGLT2i), glucagon-like peptide-1 receptor agonists (GLP-1RA), and mineralocorticoid receptor antagonists (MRAs), all of which have shown renoprotective and cardiovascular benefits. An incomplete block of aldosterone activity remains a challenge and is one of the factors contributing to the progression of kidney damage. Aldosterone synthase inhibitors (ASIs), such as vicadrostat, may represent a new horizon in selectively inhibiting aldosterone synthesis while preserving cortisol production. Early-phase trials have shown reductions in albuminuria and a potential for renal protection. The question is, could ASIs emerge as a fifth pillar in CKD management and help curb the progression?

## Introduction and background

Chronic kidney disease (CKD) affects more than 850 million people worldwide and remains a major public health concern due to its progressive nature, association with high morbidity and mortality, and high healthcare costs. CKD often leads to end-stage kidney disease requiring dialysis or transplantation, imposes a significant burden on quality of life, and frequently coexists with other chronic conditions. Among these, type 2 diabetes mellitus (T2DM) is the leading cause of CKD globally. According to the Centers for Disease Control, one in three people with T2DM also has CKD [1]. The combination of T2D and CKD drastically raises the risk of all-cause mortality, cardiovascular mortality, and cardiovascular morbidity (including myocardial infarction, strokes, peripheral artery disease, and heart failure hospitalizations), as well as the progression of kidney disease to failure [1]. In the USA, 13% of adults over 18 have diabetes, and 34.5% meet the criteria for prediabetes. Prediabetes is now being seen more often as an early warning sign for CKD because of its ties to metabolic issues and early kidney changes. This makes early intervention important to help slow or prevent progression to full-blown CKD [2,3]. Advanced stages of CKD (stages 4-5) are associated with a range of uremic symptoms, such as fatigue and pruritus, as well as complications stemming from electrolyte imbalances, including hyperkalemia, metabolic acidosis, and hypocalcemia [4,5]. As the kidney function worsens, tubular and glomerular hypertrophy, sclerosis, and fibrosis arise, leading to reductions in estimated glomerular filtration rate (eGFR), severe albuminuria, and, ultimately, kidney failure. Besides the potential for kidney death, patients diagnosed with CKD have an increased risk of developing cardiovascular-related complications before reaching end-stage kidney disease [5]. A meta-analysis including 1.4 million individuals demonstrated an increase in cardiovascular-related mortality even if they were in stage 2 CKD or had an eGFR <90 mL/min/1.73 m² [5]. For better management of CKD and to improve patient care, the National Kidney Foundation, Kidney Diseases Outcomes Quality Initiative (KDOQI), and the international guideline group Kidney Disease Improving Global Outcomes (KDIGO) developed a classification system for CKD based on eGFR and albuminuria [6-8].

According to the KDIGO guidelines, there are six eGFR stages that characterize CKD [6-8]. An estimated GFR below 60 mL/min over three months indicates impaired renal function, with severity increasing as eGFR decreases. CKD stages 1 and 2 are characterized by an eGFR of ≥90 and 60-89 mL/min/1.73 m², respectively, with evidence of kidney damage (e.g., albuminuria or structural abnormalities). Moderate CKD includes stages 3a (eGFR: 45-59 mL/min/1.73 m²) and 3b (eGFR: 30-44 mL/min/1.73 m²), representing progressive worsening in kidney function. Advanced CKD consists of stage 4 (eGFR: 15-29 mL/min/1.73 m²) and stage 5 (eGFR: <15 mL/min/1.73 m² or end-stage kidney disease), indicating significant kidney impairment or failure [6-8]. Albuminuria is classified into three categories based on the albumin-to-creatinine ratio (ACR). Albuminuria is classified into three categories: A1 (normal to mildly increased, <30 mg/g), A2 (moderately increased, 30-300 mg/g, previously termed microalbuminuria), and A3 (severely increased, >300 mg/g, formerly macroalbuminuria), with higher levels correlating with worse renal and cardiovascular outcomes [9,10]. Both eGFR and albuminuria independently predict adverse outcomes, with their combination increasing this risk further [9]. Albuminuria is a key component of CKD staging and a major independent predictor of cardiovascular events and renal progression, reflecting ongoing glomerular injury and systemic endothelial dysfunction [9]. Managing CKD becomes more complicated in individuals with T2D. The management of cardiovascular and kidney disease with T2D is based largely on lifestyle modifications, such as quitting smoking, reasonably controlling glycemia, reducing blood pressure below 130/80, and controlling lipids with statins. Limit sitting, increase stepping, improve sleep quality and duration, aim for at least 150 minutes of sweating per week, enhance strength with resistance exercises, and reduce sarcopenia are also recommended [10,11].

**Figure 1:**
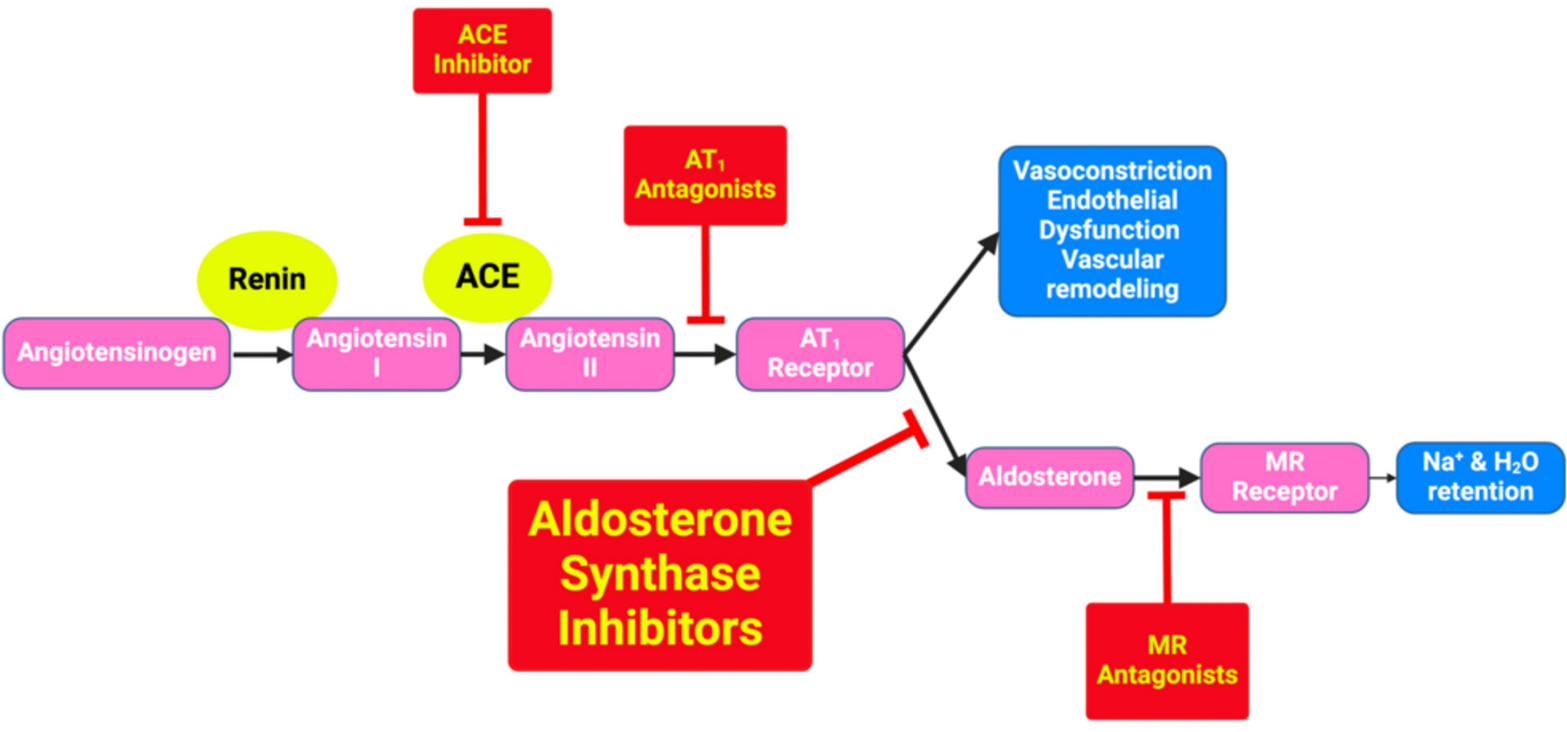
The RAAS regulates blood pressure and fluid balance. ACE inhibitors reduce angiotensin II synthesis; ARBs block AT₁ receptors; MRAs inhibit aldosterone at its receptor; and ASIs selectively inhibit aldosterone synthase (CYP11B2), directly reducing aldosterone production. ACE: Angiotensin-converting enzyme; AT1: Angiotensin type 1 receptor (AT1); ASIs: Aldosterone synthase inhibitors; MR: Mineralocorticoid receptor; RAAS: renin-angiotensin-aldosterone system Image credit: Andrew M. Treihaft

Renin-angiotensin system inhibitors (RASi) have long been established as a treatment for CKD [4]. Recently, sodium-glucose cotransporter 2 inhibitors (SGLT2i) have been proven to be effective in reducing the emergence of kidney failure when combined with RAS inhibition in a diverse population of patients [10,12,13]. It is important to note a phenomenon known as "aldosterone breakthrough" observed in many patients undergoing long-term RAS inhibition. The phenomenon is the persistence or re-occurrence of elevated aldosterone levels despite continued therapy with angiotensin-converting enzyme inhibitors or angiotensin receptor blockers. Aldosterone breakthrough contributes to ongoing sodium retention, inflammation, fibrosis, and cardiovascular remodeling, thereby diminishing the long-term reno-protective benefits of RAS inhibitors [10,14,15]. Aldosterone synthase inhibitors, such as vicadrostat, are currently being studied for their therapeutic effects by selectively targeting the enzyme aldosterone synthase, which catalyzes the final steps in aldosterone biosynthesis within the adrenal glands [10,16]. By inhibiting aldosterone synthase activity, these agents disrupt the conversion of corticosterone to aldosterone, thereby attenuating aldosterone production more directly than RAS inhibitors [1,17,18], as shown in Figure 1.

## Review

Pillars of management

**Figure 2:**
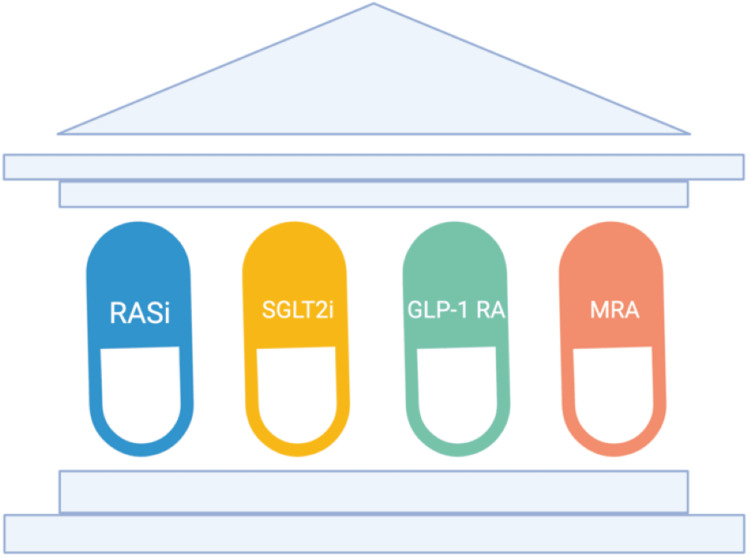
The four foundational pillars of CKD management—RAS inhibitors, SGLT2 inhibitors, GLP-1 receptor agonists, and MRAs—act synergistically to reduce intraglomerular pressure, provide cardio-renal protection, and attenuate inflammation and fibrosis. CKD: Chronic kidney disease; RASi: Renin-angiotensin system inhibitors; GLP-1RA: Glucagon-like peptide-1 receptor agonist; MRA: mineralocorticoid receptor antagonist Image credit: Andrew M. Treihaft

In managing CKD, typically, there are four key medication classes: RAS inhibitors, SGLT2 inhibitors, GLP-1RA, and MRAs, as shown in Figure 2.

RAS inhibitors, such as angiotensin-converting enzyme (ACE) inhibitors and angiotensin II receptor blockers (ARBs), help control blood pressure and reduce proteinuria, slowing kidney damage. Emerging evidence suggests that SGLT2 inhibitors mitigate kidney failure and cardiovascular risk but also retard CKD progression, irrespective of diabetic status [8]. Diuretics aid in managing fluid retention and hypertension, while statins target dyslipidemia, collectively forming the primary pharmacological arsenal to manage CKD and its associated complications [9].

RAS Inhibitors 

RAS inhibitors have been mainstream therapies in the management of CKD, because of their ability to reduce renal and cardiovascular complications through targeted blockade of the RAS pathway [19]. ACE inhibitors and ARBs effectively suppress the production or action of angiotensin II, a potent vasoconstrictor and stimulator of aldosterone release. By reducing intraglomerular pressure, diminishing proteinuria, and attenuating renal fibrosis, RAS inhibitors help preserve renal function and delay CKD progression [19]. By reducing intraglomerular pressure through dilation of the efferent arterioles, RAS inhibitors lower the mechanical stress on the glomerular capillaries, thereby diminishing proteinuria and reducing ongoing glomerular injury [19]. They also confer cardiovascular benefits by lowering BP and reducing adverse cardiovascular events in CKD patients [20]. Oparil et al. provided detailed insights into the treatment of hypertension, highlighting the role of RAS inhibitors. In short, ACEs and ARBs are able to block the production or action of angiotensin II, which is a potent vasoconstrictor [21]. By blocking angiotensin II, RAS inhibitors are able to reduce peripheral resistance, thereby lowering systemic blood pressure [21]. With the stabilization and reduction of blood pressure, ACEs and ARBs have been shown to prevent end-organ damage [21].

However, RAS inhibitors may need other agents to combat hyperkalemia. Newer potassium binders, such as patiromer and sodium zirconium cyclosilicate (SZC), have significantly enhanced the safety profile of RAS inhibitors. Patiromer and SZC act on the gastrointestinal tract by binding to potassium, which is then excreted in feces and thereby not further absorbed into the body [22]. With less potassium being absorbed, serum potassium decreases [22]. By lowering serum potassium levels, these agents reduce the risk of hyperkalemia that can occur with RAS inhibition, especially in patients with reduced renal function. With this result, they safely help providers use RAS inhibitors at therapeutic doses [22].

SGLT2 Inhibitors 

SGLT2 inhibitors, also known as flozins, are a class of medications that target the kidneys to lower blood glucose levels [23]. These inhibitors block the SGLT2 protein in the proximal convoluted tubule (PCT), blocking the reabsorption of 90% of the filtered glucose from the renal tubular lumen back into the bloodstream [23,24]. With the inhibition of SGLT2, less glucose is reabsorbed, causing glucosuria [16]. This action reduces plasma glucose, which aids in better control of T2DM. SGLT2 inhibitors also reduce sodium reabsorption through a natriuretic effect [24]. SGLT2 inhibitors have shown significant cardiovascular and renal benefits beyond glycemic control [25]. By reducing plasma volume, blood pressure, and potential direct renal effects, patients diagnosed with both CKD and heart failure have improved outcomes in morbidity and mortality [25,26]. These benefits are linked to various mechanisms, such as improved renal hemodynamics through reduced intraglomerular pressure, modulation of inflammatory markers and adipokines, and enhanced insulin sensitivity. Furthermore, SGLT2 inhibitors decrease albuminuria and slow CKD progression by reducing renal fibrosis and inflammation [27,28]. The primary cardiovascular outcome trials demonstrated the effectiveness of SGLT2 inhibitors in reducing CV mortality and hospitalizations due to heart failure among individuals with T2DM, and secondary findings from these initial trials also indicated a potential up to 40% decrease in the risk of kidney disease progression [26,28]. In the earlier cardiovascular outcome trials, most patients had relatively preserved renal function and very little albuminuria and, in more recent dedicated renal trials - such as CREDENCE, DAPA-CKD, and EMPA-KIDNEY - have included patients with more advanced CKD. In particular, EMPA-KIDNEY enrolled patients with an eGFR as low as 20 mL/min/1.73 m², and approximately 48% of participants had normoalbuminuria at baseline, demonstrating that SGLT2 inhibitors provide renal and cardiovascular benefits even in patients with low eGFR and without significant albuminuria [26,28]. CREDENCE, the pioneering double-blind, placebo-controlled randomized controlled trial (RCT), investigated the efficacy of an SGLT2 inhibitor in 4,401 participants with T2DM and CKD [28]. It showed a reduction in the risk of kidney failure or death from kidney or CV causes with canagliflozin treatment [27,28]. The DAPA-CKD trial, involving 4,304 participants with CKD and albuminuria, similarly showed the effectiveness of dapagliflozin in slowing kidney disease progression and reducing mortality from renal or CV causes [28]. The EMPAG-KIDNEY trial, a recent large randomized, double-masked, placebo-controlled trial, further reinforced the organ-protective benefits of SGLT2 inhibitors in patients with lower eGFR levels or higher albuminuria, regardless of diabetic status [27]. However, adverse events such as diabetic ketoacidosis and lower-limb amputation were slightly more frequent in the treatment group. Meta-analyses after confirmed these findings, highlighting the efficacy of SGLT2 inhibitors in reducing kidney disease progression and CV events across various patient populations, irrespective of diabetes status or underlying kidney disease [28].

Glucagon-Like Peptide-1 (GLP-1) Receptor Agonists (GLP-1 RAs)

GLP-1 RAs have become important agents in managing CKD due to their multifaceted mechanisms of action [29,30]. They bind to GLP-1 receptors on pancreatic beta cells, stimulating insulin secretion in a glucose-dependent manner, thereby improving glycemic control. Beyond glycemic management, GLP-1 receptor agonists directly affect the kidneys through several pathways [29]. They enhance natriuresis and diuresis - primarily mediated by proximal tubular inhibition - which reduces intraglomerular pressure and albuminuria, thereby mitigating renal hyperfiltration and protecting against glomerular injury [30]. GLP-1 RAs inhibit inflammatory pathways and oxidative stress, therefore adding further protection to renal anti-inflammatory and antioxidant effects [29]. Recent studies have highlighted the clinical significance of GLP-1 RAs in improving renal outcomes in patients with CKD, showing their potential as therapeutic agents beyond glycemic control alone [29].

Mineralocorticoid Receptor Antagonists (MRA)

Non-steroidal MRAs represent a novel class of agents showing promise in managing CKD by targeting the mineralocorticoid receptor (MR) without exhibiting the adverse effects of steroidal MRAs [30,31]. These agents, such as finerenone, selectively block MR activation, modulating sodium and water balance, reducing inflammation, and attenuating fibrosis in the kidney and cardiovascular system [31]. Compared to earlier MRAs, finerenone is associated with a lower risk of hyperkalemia than spironolactone and has a longer half-life and greater evidence base in CKD populations than eplerenone [31]. This selective antagonism leads to decreased expression of profibrotic and proinflammatory mediators, ultimately preserving renal function and cardiovascular health; however, while finerenone has excellent affinity for the receptor, proceed with caution with medications that can induce the cytochrome p450 enzyme 3A4 [31]. It is approved for diabetic patients with kidney disease. Aldosterone antagonists do not block non-genomic receptors, limiting their success. Aldosterone antagonists lower blood pressure but reactively increase renin and aldosterone levels [31].

Statins 

Statins have gained attention for their potential benefits in CKD beyond lipid-lowering effects, primarily due to their anti-inflammatory properties. By inhibiting HMG-CoA reductase, statins reduce cholesterol synthesis and have anti-inflammatory effects that are particularly relevant in CKD, where inflammation plays a significant role in disease progression [32]. Studies have demonstrated that statin therapy in CKD patients can lower levels of inflammatory markers, such as C-reactive protein, and attenuate endothelial dysfunction, potentially slowing renal deterioration and reducing cardiovascular risk [32]. However, in advanced CKD, the use of statins, particularly in dialysis-dependent patients (eGFR <30 mL/min/1.73 m²), remains controversial. While large trials such as 4D and AURORA have not demonstrated significant cardiovascular benefit in this population, statins may still be considered for primary or secondary prevention in selected patients, particularly those not yet on dialysis [33,34].

Aldosterone Synthase Inhibitors 

**Figure 3:**
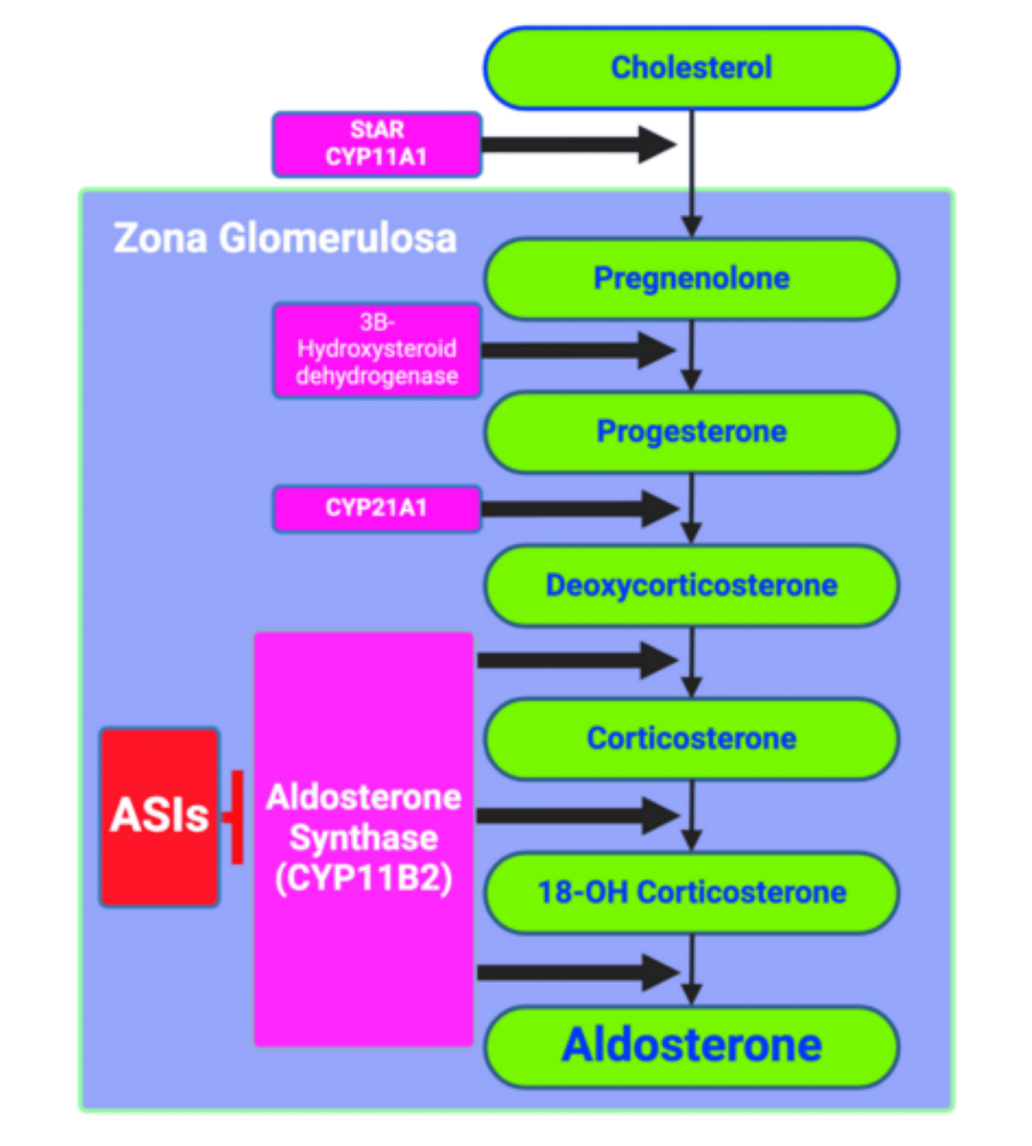
ASIs are a new class of drugs manufactured to selectively inhibit aldosterone synthase (CYP11B2), an important enzyme catalyzing the final steps of aldosterone synthesis in the zona glomerulosa of the adrenal gland. With the selectivity, these inhibitors are effective in the reduction of aldosterone without affecting cortisol synthesis. ASI: Aldosterone synthase inhibitor Image credit: Andrew M. Treihaft

Patients have demonstrated beneficial outcomes with the advent of MRAs, such as spironolactone and eplerenone conditions with heart failure and proteinuric nephropathy [35]. MRAs, however, do have their side effect profiles, such as increased levels of renin and aldosterone, potentially decreasing their long-term efficacy. Aldosterone affects both genomic (via MR activation) and non-genomic activity, and MRAs do not completely block aldosterone activity due to the similarities between aldosterone synthase (CYP11B2) and cortisol synthase (CYP11B1) [10]. To combat these limitations, a novel medication class, aldosterone synthase inhibitors (ASIs), has been manufactured to inhibit the direct biosynthesis of aldosterone while decreasing off-target effects, as demonstrated in Figure 3.

The first oral ASI, osilodrostat, demonstrated effective plasma urinary aldosterone; however, it lacked sensitivity at higher doses, leading to inhibition of cortisol production. With the inhibition of cortisol, the FDA approved osilodrostat for the treatment of Cushing's disease. However, it was not approved for CKD management [10,36-38]. After further reiterations of ASIs, a new ASI, vicadrostat was developed and is currently being investigated for CKD treatment. Preclinical and early-phase trials demonstrated that vicadrostat hinders aldosterone production without affecting cortisol synthesis, showing a promising path for managing diabetic nephropathy. Tuttle et al.'s phase 2 trial evaluated patients diagnosed with CKD on ACEis or ARBs with or without SGLT2is to receive vicadrostat [10]. The study concluded that there was a significant decrease in the urine albumin-to-creatine ratio, supporting its potential renal benefits. The phase 3 trial is currently underway and is evaluating long-term efficacy in CKD progression, as well as cardiovascular risk reduction. The study was designed to evaluate patients diagnosed with or without T2DM to receive empagliflozin or placebo, followed by vicadrostat or placebo, and further investigate its impact on renal function [10].

The future

In clinical practice, CKD remains a significant burden in part due to its nature of progression and association with cardiovascular morbidity and mortality. With the ever-rising prevalence of CKD, especially for patients with T2DM, medical providers need to approach disease management that balances established and emerging therapies for their patients. The current management of CKD involves primarily controlling blood pressure, reducing albuminuria, and reducing cardiovascular risk through medications such as RAS inhibitors, SGLT2is, GLP-1RA, and MRAs [11]. Although effective, there is still incomplete blockage of the renin-angiotensin system, which leads to worsening effects on the body by aldosterone's nongenomic actions, such as inflammation and fibrosis of the kidney [10,36]. ASIs are an emerging class of therapeutics manufactured to suppress aldosterone production without the side effects of MRAs, such as hyperkalemia [35]. Vicadrostat is gaining interest due to its selective inhibition of aldosterone synthesis with the preservation of cortisol production [10]. The Tuttle investigation with CKD patients receiving standard care therapies, i.e., RASi and SGLT2i, demonstrated key reductions in the urine-to-creatinine ratio, suggesting possible additional renal protection with existing therapies [11]. The reduction in albuminuria observed with vicadrostat in the phase 2 trial by Tuttle et al. was approximately 39.5% from baseline in the treatment group. This reduction was statistically significant (p<0.01) compared to placebo [10,15]. With the phase 3 trial underway, the medical community can follow along to see if vicadrostat has an efficacious long-term effect in reducing CKD progression and cardiovascular outcomes [10]. It is important to note that vicadrostat remains under investigation and has not yet demonstrated superiority over MRAs in terms of long-term renal or cardiovascular outcomes. The ongoing phase 3 trial (NCT05536803) is expected to be completed by 2026 and will provide further insights into its safety and efficacy profile [16].

With advances in medicine, introducing novel agents such as vicadrostat into clinical practice could enhance CKD management by targeting direct pathways that were not addressed. Further research will help determine how ASIs may complement or compete with existing therapy - such as SGLT2 inhibitors and MRAs - in regulating renal hemodynamics, fibrosis, and inflammation while reducing risks such as hyperkalemia. Continuing to explore these potential synergies will be essential to optimizing individualized treatment strategies for patients diagnosed with CKD [10].

## Conclusions

CKD poses a significant public health problem, especially with patients diagnosed with T2DM, given the increased association of cardiovascular risks. The progression of CKD leads to increased morbidity and mortality, demonstrating a need for early detection and effective management. The stages of CKD based on eGFR and albuminuria provide a framework for risk assessment and treatment. The four pillars of medications for managing CKD, RASi, SGLT2i, GLP-1RA, and MRA have all demonstrated renal and cardiovascular protective benefits. With the investigation of ASIs such as vicadrostat, an emerging fifth pillar may be in construction as a therapy targeting aldosterone-driven CKD progression. Even with advancements in therapy, CKD remains a progressive condition requiring a multifactorial intervention such as lifestyle modifications and pharmacological interventions. Continuing to research ASIs may further improve outcomes for patients diagnosed with CKD, and it is crucial to know that moving forward and optimizing individual strategies may reduce CKD-related morbidity and mortality.
